# Structure of the unique tetrameric STENOFOLIA homeodomain bound with target promoter DNA

**DOI:** 10.1107/S205979832100632X

**Published:** 2021-07-29

**Authors:** Prabhat Kumar Pathak, Fei Zhang, Shuxia Peng, Lifang Niu, Juhi Chaturvedi, Justin Elliott, Yan Xiang, Million Tadege, Junpeng Deng

**Affiliations:** a Department of Biochemistry and Molecular Biology, Oklahoma State University, Stillwater, OK 74078, USA; b Department of Plant and Soil Sciences, Institute for Agricultural Biosciences, Oklahoma State University, Ardmore, OK 73401, USA; c Department of Microbiology and Immunology, University of Texas Health Science Center at San Antonio, San Antonio, TX 78229, USA

**Keywords:** homeobox transcription factors, homeodomains, STENOFOLIA, WUSCHEL, WOX, crystal structure, *Medicago truncatula*

## Abstract

Homeobox transcription factors are key regulators of morphogenesis and development in both animals and plants. In plants, the WUSCHEL-related homeobox (WOX) family of transcription factors function as central organizers of several developmental programs ranging from embryo patterning to meristematic stem-cell maintenance through transcriptional activation and repression mechanisms. The *Medicago truncatula* *STENOFOLIA* (*STF*) gene is a master regulator of leaf-blade lateral development. Here, the crystal structure of the homeodomain (HD) of STF (STF-HD) in complex with its promoter DNA is reported at 2.1 Å resolution. STF-HD binds DNA as a tetramer, enclosing nearly the entire bound DNA surface. The STF-HD tetramer is partially stabilized by docking of the C-terminal tail of one protomer onto a conserved hydrophobic surface on the head of another protomer in a head-to-tail manner. STF-HD specifically binds TGA motifs, although the promoter sequence also contains TAAT motifs. Helix α3 not only serves a canonical role as a base reader in the major groove, but also provides DNA binding in the minor groove through basic residues located at its C-terminus. The structural and functional data *in planta* reported here provide new insights into the DNA-binding mechanisms of plant-specific HDs from the WOX family of transcription factors.

## Abstract 2

The STENOFOLIA homeodomain binds promoter DNA as a tetramer and the α3 helix binds DNA in both the major and minor grooves.

## 1. Introduction   

Homeodomain (HD)-containing transcription factors are some of the most important regulators of morphology and differentiation in fungi, animals and plants (Garcia-Fernàndez, 2005 ▸; Miksiunas *et al.*, 2020 ▸; Bürglin & Affolter, 2016 ▸). Among the various types of HD-containing transcriptional regulators, the WUCHEL-related homeobox (WOX) family is unique to plants (Zhou *et al.*, 2015 ▸; Mayer *et al.*, 1998 ▸; Sarkar *et al.*, 2007 ▸; Mukherjee *et al.*, 2009 ▸) and instructs plant growth and development from a small group of pluripotent cells analogous to the stem-cell niche in animals. *WOX* genes play central roles in apical–basal polarity patterning during embryogenesis and in maintaining the stem-cell niches in various plant meristems during post-embryonic shoot and root growth and the development of lateral organs such as leaves and flowers (Costanzo *et al.*, 2014 ▸; Yadav *et al.*, 2011 ▸; Han *et al.*, 2020 ▸; Hao *et al.*, 2019 ▸; Jha *et al.*, 2020 ▸; Kieffer *et al.*, 2006 ▸; Laux *et al.*, 1996 ▸).

WUSCHEL (WUS), the founding member of the WOX family, is a conserved key regulator for shoot apical meristem (SAM) and axillary meristem (Mayer *et al.*, 1998 ▸; Kieffer *et al.*, 2006 ▸; Meng *et al.*, 2019 ▸; Stuurman *et al.*, 2002 ▸; Wang *et al.*, 2019 ▸). WUS paralogs perform similar functions, including WOX5 in root apical meristem (Sarkar *et al.*, 2007 ▸), WOX4 in procambial/cambial meristem (Hirakawa *et al.*, 2010 ▸; Ji *et al.*, 2010 ▸), and WOX1 and WOX3 in leaf marginal meristem (Nakata *et al.*, 2012 ▸; Vandenbussche *et al.*, 2009 ▸). The *Medicago truncatula WOX1* gene, *STENOFOLIA* (*STF*), and its *Nicotiana sylvestris* ortholog, *LAMINA1* (*LAM1*), regulate leaf-blade outgrowth by promoting cell proliferation at the adaxial–abaxial junction through transcriptional repression (Tadege *et al.*, 2011 ▸; Lin *et al.*, 2013 ▸; Zhang *et al.*, 2014 ▸). WUS clade WOX members have a promiscuous ability to substitute for each others’ function if driven by specific promoters, as demonstrated by the complementation of the *lam1* mutant in leaf development (Lin, Niu, McHale *et al.*, 2013 ▸) and of the *wus* mutant in SAM maintenance (Dolzblasz *et al.*, 2016 ▸), suggesting a conserved mechanism of DNA recognition and transcriptional repression. WUS clade members, including WUS and WOX1–WOX7, share a conserved WUS box at the C-terminus that is specific to the WUS clade (Haecker *et al.*, 2004 ▸; Ikeda *et al.*, 2009 ▸; Lin, Niu, McHale *et al.*, 2013 ▸) and a conserved HD that is typical of the whole WOX family (Costanzo *et al.*, 2014 ▸). While the HD contacts DNA, the WUS box is essential for the recruitment of the TOPLESS (TPL) family of transcriptional co-repressors (Busch *et al.*, 2010 ▸; Zhang *et al.*, 2014 ▸). Both the WUS box and HD are essential for the roles of STF in leaf development and of WUS in shoot meristem maintenance.

The HD has a canonical structure that mainly comprises a three-α-helical bundle and is found in a large class of transcription factors that are ubiquitous in fungi, animals and plants (Bürglin & Affolter, 2016 ▸). HD-containing transcription factors share low sequence identity and recognize variable DNA sequences (Noyes *et al.*, 2008 ▸). A typical HD is about 60 amino acids in length, but several types of atypical HD proteins have more or fewer residues (Bürglin, 1997 ▸; Tadege *et al.*, 2011 ▸). HDs of the WOX family contain about 65–70 residues.

WUS functions by binding to at least two distinct DNA motifs: the G-box motif (TCACGTGA) and the TAAT motif [TTAAT(G/C)(G/C)] (Busch *et al.*, 2010 ▸; Yadav *et al.*, 2011 ▸; Lohmann *et al.*, 2001 ▸). It has also been reported that WUS can bind to TGAA repeats (O’Malley *et al.*, 2016 ▸; Sloan *et al.*, 2020 ▸). Besides these WUS binding sites, STF can also bind strongly to (GA)/(CT)_*n*_ elements (Liu *et al.*, 2018 ▸). Although HDs from other kingdoms of life have been well characterized structurally (Bürglin & Affolter, 2016 ▸; Fraenkel & Pabo, 1998 ▸; Lu *et al.*, 2007 ▸; Miyazono *et al.*, 2010 ▸; Passner *et al.*, 1999 ▸; Wolberger *et al.*, 1991 ▸; Li *et al.*, 1995 ▸; Lee *et al.*, 2018 ▸; Zhang *et al.*, 2011 ▸), the structures and DNA-binding mechanisms of the HDs from WOX family members have been less well studied.

HD-containing transcription factors have been shown to form homodimers and heterodimers in DNA binding (Busch *et al.*, 2010 ▸; Nagasaki *et al.*, 2005 ▸; Bürglin & Affolter, 2016 ▸). The protein–protein interactions between homodimers and heterodimers of HD proteins allow the transcription factors to recognize different DNA sequences longer than four base pairs, activating genes in a selective manner *in vivo* (Bürglin & Affolter, 2016 ▸). Recently, structures of the WUS homedomain (WUS-HD) in complex with three DNA sequences containing distinct motifs have been reported (Sloan *et al.*, 2020 ▸), which showed a dynamic dimeric HD–DNA binding mode. Here, we report the crystal structure of the STF homeodomain in complex with its target promoter DNA containing both TGA and TAAT motifs, providing new insights into the mechanism by which WOX family transcription factors recognize DNA.

## 2. Materials and methods   

### 2.1. Protein purification and crystallization   

The coding sequence of *M. truncatula* STENOFOLIA residues 85–190 (STF-HD; gene ID JF276252.1) was amplified by PCR and inserted into a modified pET vector as an MBP fusion with an N-terminal 6×His tag that is cleavable by Tobacco etch virus (TEV) protease. The recombinant protein was expressed in *Escherichia coli* and purified by Ni–NTA affinity purification procedures as described previously (Krumm *et al.*, 2008 ▸). Briefly, STF-HD protein was first purified from the soluble cell lysate on an Ni–NTA affinity column using loading buffer (20 m*M* Tris–HCl, 500 m*M* NaCl, 20 m*M* imidazole pH 8.0). The protein was eluted with elution buffer (loading buffer plus 250 m*M* imidazole) and subsequently subjected to TEV protease cleavage at a 1:100 mass ratio while dialyzing against loading buffer at 4°C overnight. The protein was then collected as the flowthrough from a second subtracting Ni–NTA column. The protein was further purified by size-exclusion chromatography to homogeneity in a buffer consisting of 20 m*M* Tris–HCl pH 7.4, 125 m*M* NaCl, 5 m*M* tris(2-carboxyethyl)phosphate (TCEP). The STF-HD triple mutant (L107M/L110M/L130M) was cloned using the PCR-based site-directed mutagenesis method. The selenomethionine (SeMet)-substituted triple-mutant and wild-type (WT) proteins were expressed in *E. coli* BL21(DE3) cells with SeMet-supplemented M9 medium. All STF-HD mutant proteins and SeMet-substituted proteins were purified using the same procedures as described above. The purified proteins were concentrated to 20–25 mg ml^−1^, flash-frozen in liquid nitrogen and stored at −80°C until use (Deng *et al.*, 2004 ▸).

The 22 bp synthetic oligonucleotide containing the sequence 5′-GCAAATTAATGATTTATTCAAG-3′ and its complementary strand 5′-CTTGAATAAATCATTAATTTGC-3′ (*MtLOB39*; Supplementary Table S1) were purchased from Integrated DNA Technologies (IDT) and annealed at 1 m*M* concentration in a buffering solution consisting of 50 m*M* HEPES pH 7.2, 50 m*M* NaCl, 5 m*M* magnesium chloride with a descending temperature gradient from 95°C to 23°C in 2 h. The purified STF-HD proteins were mixed with the annealed 22 bp dsDNA in a 4:1 molar ratio and stored on ice for 30 min before crystallization trials. The sitting-drop vapor-diffusion method was used in the crystallization trials by mixing 0.5 µl protein–DNA complex solution with 0.5 µl reservoir solution. All crystals were obtained from a condition consisting of 0.15 *M* NaCl, 28%(*v*/*v*) polyethylene glycol (PEG) Smear Medium (Chaikuad *et al.*, 2015 ▸) at 20°C. 20% glycerol was added to the mother liquor as a cryoprotectant before flash-cooling the crystals in liquid nitrogen.

### 2.2. Structure determinations   

All data were collected on beamline 19-ID at the Advanced Photon Source (APS), Argonne National Laboratory. Our attempts to use the molecular-replacement method to solve the native data set using canonical HD-domain structures as a template failed as the structure of WUS-HD was not available at the time. SeMet-substituted WT STF-HD crystals did not yield usable anomalous signal to solve the structure due to the disorder of the single Met160 present in the protein. Based on homology modeling with HD domains, we made a triple mutant of STF by substituting three buried leucine residues with methionines (L107M/L110M/L130M). The structure of the STF-HD–DNA complex was solved by the single-wavelength anomalous dispersion method using *HKL*-3000 (Minor *et al.*, 2006 ▸) with data collected from a single SeMet-substituted triple-mutant protein crystal. 70% of all protein residues were constructed from the experimental phases obtained from the SeMet-substituted crystal data using *AutoBuild* in *Phenix* (Liebschner *et al.*, 2019 ▸). The remaining residues and the 22 bp dsDNA were built manually using *Coot* (Emsley *et al.*, 2010 ▸). This model was used to solve the native structure at higher resolution by the molecular-replacement method using *Phaser* (McCoy, 2007 ▸). *Phenix* was used for the refinement and *Coot* was used for iterative manual model building. The translation, libration and screw-rotation displacement (TLS) groups used in the refinement were defined by the *TLMSD* server (Painter & Merritt, 2006 ▸). The final *R*_work_ and *R*_free_ values for the refined model were 19.4% and 25.0%, respectively. The current model has good geometry and refinement statistics (Table 1 ▸). Electrostatic surface potentials were determined using *PDB*2*PQR* (Dolinsky *et al.*, 2004 ▸, 2007 ▸) and the *APBS* (Baker *et al.*, 2001 ▸) plugin in *PyMOL* (DeLano, 2002 ▸), and were visualized over the range −4 *kT* e^−1^ to +4 *kT* e^−1^. All molecular-graphics figures were generated with *PyMOL*.

### 2.3. EMSA for protein–DNA binding   

To optimize the protein:DNA ratio used for crystallization, the binding of DNA to the STF-HD protein was analyzed by titration using an agarose gel-based electrophoretic mobility shift assay (EMSA). 5′ 6-FAM-labeled 22 bp DNA oligo­nucleotides (from IDT) with sequences from the *MtLOB39* and *MtAS2* promoters (Supplementary Table S1) were mixed with the purified proteins in various molar ratios and incubated on ice for 60 min before electrophoresis on 1% agarose gels in 1× TAE buffer for 60 min at 90 V at 4°C. The gels were subsequently analyzed on a Bio-Rad ChemiDoc fluorescence imager with excitation and emission wavelengths of 497 and 520 nm, respectively, to visualize the DNA. The gels were also stained with Coomassie Blue for protein detection. From these analyses, it was determined that mixing the protein with DNA in a 4:1 molar ratio would readily form a stable complex, and this ratio was therefore used in all crystallization trials.

Comparisons of DNA binding by the WT and mutant STF-HD proteins were analyzed by native polyacrylamide gel-based EMSA using a previously described method (Zhang *et al.*, 2010 ▸). Briefly, oligonucleotides were synthesized with the 3′ Biotin CPG modification. Oligonucleotides were annealed and incubated with His-MBP, His-MBP-STF^85–190^ or His-MBP-STF^85–190^ mutant fusion proteins using the Light Shift Chemiluminescent EMSA Kit (Pierce) at room temperature for 30 min. The binding reaction consisted of 10 m*M* Tris–HCl pH 7.5, 100 m*M* KCl, 1 m*M* DTT, 2.5% glycerol, 5 ng µl^−1^ poly(dI·dC), 0.05% NP-40, 0.05 µg µl^−1^ purified protein and 5 fmol µl^−1^ annealed oligonucleotides. Gel electrophoresis was performed on a 5% native polyacrylamide gel at 100 V for 45 min at room temperature. After blotting on a positively charged nylon membrane, the DNA was cross-linked using a transilluminator under standard conditions. The biotin-labeled DNA was then detected using the Chemiluminescent Nucleic Acid Detection Module Kit (Pierce).

### 2.4. Fluorescence polarization assays   

The fluorescence polarization (FP) assays were conducted using a BMG Labtech Pherastar FS multimode plate reader, with an excitation wavelength of 485 nm and an emission wavelength of 520 nm. The 5′ 6-FAM-labeled dsDNA (*MtLOB39*; Supplementary Table S1) was used as a probe in solution with various amounts of purified STF-HD proteins to evaluate protein–DNA interactions by monitoring the change in the polarization of the emitted light. Both the proteins and the DNA probe were in a buffering solution consisting of 50 m*M* Tris–HCl pH 7.4, 125 m*M* NaCl; each well contained 30 µl of this mixture. The DNA concentration was kept constant at 50 n*M* and the protein concentration was decreased by twofold steps in the serial dilutions. Millipolarization values from wells with only the probe were subtracted as a background from all measurements. The *K*_d_ values as an overall estimation of the binding affinities of STF-HD proteins and DNA were calculated using *Graphpad Prism* 8 by a nonlinear regression for curve fitting. Standard deviation values were derived from triplicates for each data point.

### 2.5. Plant materials and growth conditions   

The *N. sylvestris* wild type and *lam1* mutant were used in this research. Plants were grown in a controlled greenhouse with 24°C/16 h (day) and 20°C/8 h (night) photoperiods, 60–70% relative humidity and 150 µmol m^−2^ s^−1^ light intensity.

### 2.6. Plasmid construction and plant transformation   

All *lam1* complementation assays were performed using the pSTF-pMDC32 Gateway vector as described by Lin, Niu, McHale *et al.* (2013 ▸). The mutations in STF were introduced using appropriate mutagenic primers and were confirmed by sequencing (Supplementary Table S1). WT STF and STF mutants were each cloned into the pDONR207 vector and then ligated into the pSTF-pMDC32 destination vector by LR reaction (Invitrogen). The constructs were introduced into *Agrobacterium tumefaciens* strain GV2260 for *N. sylvestris* transformation. Leaf blades from two-month-old *lam1* mutants were used for transformation using the procedures described previously (Tadege *et al.*, 2011 ▸). The complementation strength was evaluated by the length/width ratio of the largest leaf in each independent transgenic line. At least ten independent lines were analyzed for each construct (Supplementary Table S2). Statistical analyses were performed using one-way ANOVA followed by a Tukey’s multiple comparisons test in *GraphPad Prism* 8, with significant differences *p* < 0.05.

## 3. Results   

### 3.1. The structure of the STF-HD–DNA complex   

**Figure 1:**
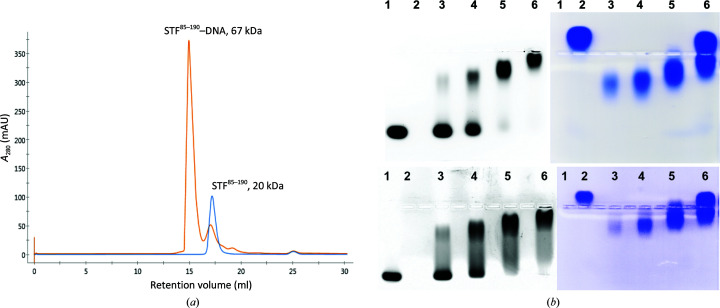
STF-HD oligomerizes upon binding DNA. (*a*) Chromatographs of the apoprotein (blue) and the DNA complex (orange) from a Superdex S200 column. The estimated molecular weight (MW) of the apo protein from the retention volume is about 20 kDa, which is larger than the theoretical MW of 12.5 kDa of STF-HD due to its elongated shape. These data are in agreement with the STF-HD monomer, which has a calculated apparent MW of 22.5 kDa in solution based on its calculated hydrodynamic radius of 23.4 Å (Fleming & Fleming, 2018 ▸) using the current crystal structure. The *A*_280_/*A*_260_ of the peak faction collected from the complex had a value of 1.61, suggesting a 1:4 DNA:protein ratio in the complex. (*b*) EMSA analysis of STF-HD binding to the promoter DNA. 5′ 6-FAM-labeled 22 bp DNA (top, *MtLOB39* promoter; bottom, *MtAS2* promoter) was used in the assay. Left (black and white): the fluorescence signal from the DNA is captured. Right: the same gel stained with Coomassie Blue to show the protein. Lane 1, 5′ 6-FAM-labeled DNA; lane 2, apoprotein; lanes 3–6, DNA and protein mixed at molar ratios of 1:1, 1:2, 1:4 and 1:8, respectively. Note that STF-HD forms a stable complex with DNA when mixed in a 1:4 molar ratio (DNA:protein) in lane 5.

**Figure 2:**
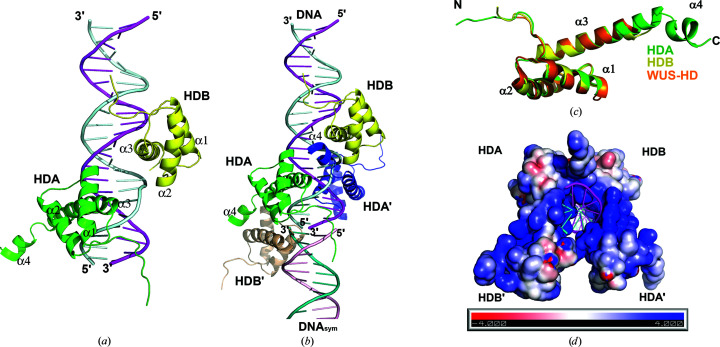
The structure of the STF-HD–DNA complex. (*a*) STF-HD dimer (HDA in green and HDB in yellow) bound to a 22 bp DNA (strands colored magenta and light cyan) in the asymmetric unit. (*b*) Four STF-HDs are depicted (HDA′ in blue and HDB′ in wheat) bound on the DNA. HDA and HDA′ and HDB and HDB′ are crystallographic symmetry mates. A second DNA molecule in the crystal packing is shown (DNA_sym_; light pink and teal) forming a pseudo-continuous helix. (*c*) Superimpositions of the structures of HDA, HDB and WUS-HD (PDB entry 6ryd). (*d*) The electropotential surface of the STF-HD tetramer is shown. The bound dsDNA is shown as cartoons colored as in (*b*). Note that nearly the entire DNA surface is clamped by the protein.

Apo STF^85–190^ protein (STF-HD) appeared as a monomer in solution (Fig. 1 ▸ *a*). However, it forms a stable complex with its target promoter DNA when mixed together in a 1:4 (DNA:protein) molar ratio (Fig. 1 ▸, Section 2). After screening a number of synthetic DNA oligonucleotides, we found that STF-HD readily crystallized when in complex with a 22 bp target promoter DNA. The structure of the complex was determined by single-wavelength anomalous dispersion using a SeMet-substituted crystal of the triple-mutant STF protein (L107M/L110M/L130M; see Section 2). There are two protein molecules and one DNA molecule in the asymmetric unit of the crystal (Fig. 2 ▸ *a*). The overall structure of STF-HD adopts the canonical HD architecture comprised of a three-α-helical bundle core connected by well ordered loops and a long arm of peptide at the N-terminus, with overall dimensions of approximately 42 × 32 × 25 Å. The two protomers HDA and HDB are positioned more than 8 Å apart and are bound on the opposite sides of the DNA, making no direct protein–protein interactions with each other. There are also two additional STF-HD molecules bound on the same DNA (HDA′ and HDB′), which are related to HDA and HDB, respectively, in the crystal via crystallographic symmetry (Fig. 2 ▸ *b*), forming a tetramer. The N- and C-termini of the protein are partially disordered and the longest visible protein chain in the current structure contains residues 89–172. The core structures of the two STF-HD protomers (HDA and HDB) adopt a nearly identical conformation, with a root-square-mean deviation (r.m.s.d.) of 0.78 Å over 66 equivalent C^α^ atoms. In both HDA and HDB, the α3 helices are significantly longer than the other helices and are perpendicular to α2, adopting a classical helix–turn–helix motif. HDA has 13 more residues clearly visible at its C-terminus, which contains a short helix α4, when compared with HDB, which has a more disordered and partially unwound C-terminus (Fig. 2 ▸ *c*).

**Figure 3:**
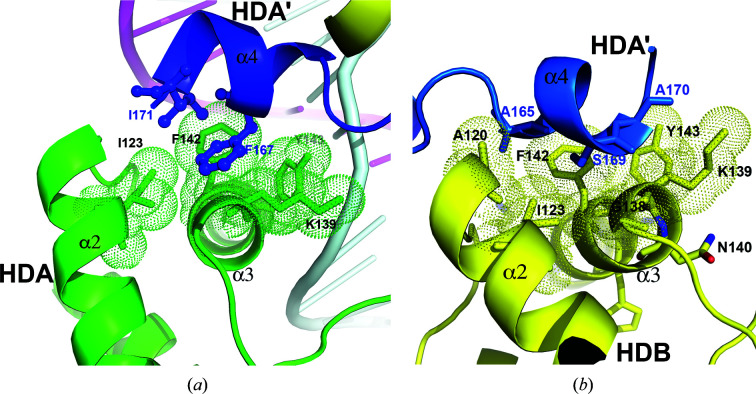
The STF-HD tetramer interface. (*a*) The interface between HDA (green) and HDA′ (blue). The contacting residues are shown as sticks, with dotted envelopes indicating the van der Waals radius. (*b*) The interface between HDA′ (blue) and HDB (yellow).

The four STF-HD molecules tightly clamp around nearly the entire surface of the DNA, spanning three grooves (Figs. 2 ▸ *b* and 2 ▸ *d*) and burying about 5435 Å^2^ of solvent-accessible surface (SAS). The protein–protein interactions among the four STF-HD molecules are mainly found to involve the ordered C-terminal α4 helices in HDA and HDA′, which bridge the tetramer together on the DNA (Figs. 2 ▸ *b* and 3 ▸). Both HDA and HDB use a common docking surface pocket to interact with helix α4 of HDA′. This docking pocket is composed of nonpolar residues located on helices α2 (Ala120 and Ile123) and α3 (Gly138, the aliphatic side chain of Lys139, Phe142 and Tyr143; Fig. 3 ▸) in both molecules. Helix α4 of HDA′ is sandwiched between HDA and HDB, with one surface involved in contact with HDA (Phe167 and Ile171; Fig. 3 ▸ *a*), burying about 442 Å^2^ of SAS, while the opposite surface is involved in docking with HDB (Ala165, Ser169 and Ala170; Fig. 3 ▸ *b*), burying about 574 Å^2^ of SAS. The HDB′–HDA interface is the same as the HDA′–HDB interface.

### 3.2. STF-HD specifically recognizes the TGA DNA sequence   

**Figure 4:**
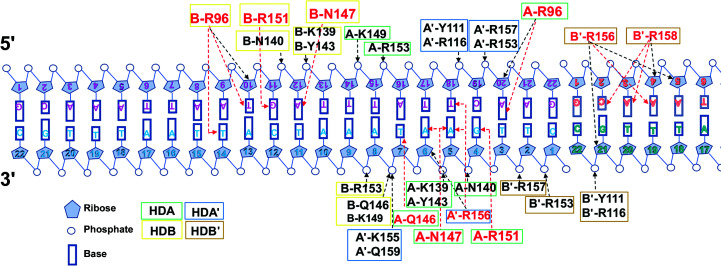
Schematic representation of interactions between STF-HD and DNA. The residues involved in base contacts are colored red and those involved in ribose and/or backbone contacts are colored black. Residues from each STF-HD are labeled and enclosed in boxes that are colored as in Fig. 2 ▸(*b*). Hydrogen bonds are shown as dashed arrows; those in red indicate base contacts and those in black indicate ribose/backbone contacts. Residues involved in nonpolar interactions are not shown.

**Figure 5:**
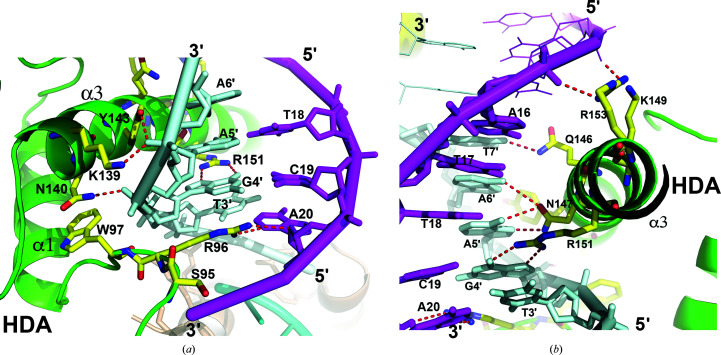
TGA recognition by HDA. (*a*) The HDA N-terminal arm interacts with the DNA in the minor groove. (*b*) HDA helix α3 contacts the DNA in the major groove. The secondary structures are shown as cartoons and colored as in Fig. 2 ▸(*a*). The contacting protein residues are shown as sticks and colored by element. Hydrogen bonds are indicated as red dashed lines.

The STF-HD tetramer interacts with the DNA extensively. HDA and HDB are bound at two TGA motifs that are arranged as an inverted repeat and are separated by five base pairs, making contacts with the DNA via both the major and minor grooves (Figs. 2 ▸ *b* and 4 ▸). In both HDA and HDB, the N-terminal arms embrace the DNA from the minor grooves, while the α3 helices are inserted into the major grooves of the DNA. Arg96 (N-terminal arm), Asn147 and Arg151 (helix α3) serve as a molecular probe for STF to read out the TGA DNA fingerprint. In HDA, Arg96 forms hydrogen bonds to the N3 and O4′ atoms of nucleoside A20, while the flanking Ser95 and Trp97 embrace the DNA via van der Waals interactions (Figs. 4 ▸ and 5 ▸ *a*). Asn147 and Arg151 recognize the G4′/A5′ step in the reverse strand (Fig. 5 ▸ *b*). While the OD1 atom of Asn147 forms bifurcated hydrogen bonds to the N6 atoms of A5′ and A6′, the ND2 atom is hydrogen-bonded to the N7 atom of base A5′. Arg151 is hydrogen-bonded to the N7 and O6 atoms of base G4′. In addition, the NE2 atom of Gln146 is hydrogen-bonded to the O4 atom of base T7′. This interaction may not be base-specific since Gln146 could potentially be hydrogen-bonded to the N4 atom of a cytosine base via its OE1 atom instead. Phe142 and Tyr143 embrace the bases, while Trp144 contacts the backbone of the DNA through van der Waals interactions. The polar or charged side chains of Lys139, Asn140, Tyr143, Lys149 and Arg153 on helix α3 also contact the DNA backbone through hydrogen bonds and salt bridges (Figs. 4 ▸ and 5 ▸).

**Figure 6:**
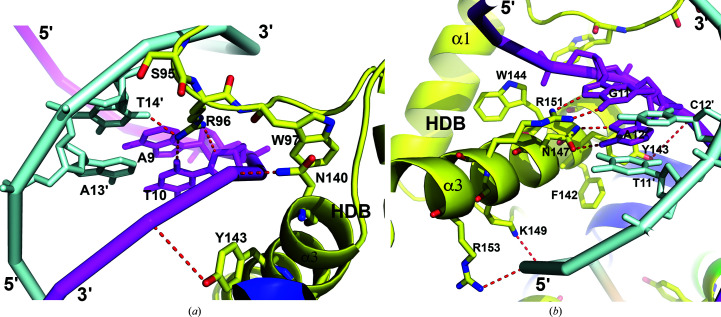
TGA recognition by HDB. (*a*) The HDB N-terminal arm interacts with the DNA in the minor groove. (*b*) HDB helix α3 contacts the DNA in the major groove. The color scheme is the same as in Fig. 2 ▸(*a*).

In HDB, Arg96 forms hydrogen bonds to the O2 and O4′ atoms of nucleoside T10 and the O2 of base T14′ on the reverse strand. Ser95 and Trp97 brace the DNA via van der Waals interactions (Fig. 6 ▸ *a*). These exquisite interactions could contribute to the DNA-binding affinity of STF-HD. Asn147 is hydrogen-bonded to the N7 and N6 atoms of base A12, while Arg151 is hydrogen-bonded to the N7 and O6 atoms of base G11 (Figs. 4 ▸ and 6 ▸ *b*). In addition to these base-specific interactions, helix α3 of HDB also contacts the backbone of the DNA via hydrogen bonds and salt bridges (Lys139, Asn140, Tyr143, Gln146, Lys149 and Arg153; Figs. 4 ▸ and 6 ▸). Furthermore, the aromatic side chains of Phe142, Tyr143 and Trp144 establish additional hydrophobic inter­actions with the DNA, as observed in HDA.

The two crystallographic symmetry-related STF-HD molecules, HDA′ and HDB′, mainly contact the DNA in the minor grooves using polar and basic residues located in the C-terminal halves of their α3 helices. In HDA′, Arg156 forms hydrogen bonds to the N3 atom of base A5′, the O4′ atom of nucleoside A6′ and the O2 atom of base T18. Arg153, Lys155, Arg157 and Gln159 contact the DNA backbone via salt bridges and hydrogen bonds (Figs. 4 ▸ and 7 ▸ *a*). In addition, Met160 located at the C-terminus of helix α3 is inserted into the minor groove of the DNA, providing van der Waals interactions. Arg116 from the α1/α2 loop and Tyr111 located on helix α1 also contact the DNA backbone through their charged or polar side chains. While its head is tethered to the tail of HDA, the C-terminus of helix α3 of HDB′ is inserted into the minor groove at the junction between two pseudo-continuous DNA molecules in the crystal packing (Figs. 2 ▸ *b*, 4 ▸ and 7 ▸ *b*). In comparison to HDA′, the C-terminus of HDB′ is more disordered, with the last visible residue being Gln159, and the end of helix α3 is unwound. Arg153 and Arg157 make contacts with the DNA backbone. Arg156 and Arg158 contact the subsequent DNA molecule in the minor groove. Arg156 forms hydrogen bonds to the N3 atom of base A4 and the O4′ atoms of nucleosides A5 and G21′, while Arg158 contacts the O2 atom of base C2, the N3 atom of base A3 and the O4′ atom of nucleoside A4. Similar to as observed in HDA′, Arg116 and Tyr111 in HDB′ make additional contacts with the DNA backbone via charged and polar side chains (Figs. 4 ▸ and 7 ▸ *b*).

To our knowledge, this is the first time that the α3 helix from an HD has been observed to bind DNA in the minor groove, since it has predominantly been observed in the recognition of specific DNA sequences in the major grooves of other HD structures.

### 3.3. Structure-based mutagenesis: key residues for DNA recognition and STF function   

The DNA-binding mode of STF-HD in the current structure is complex, since all four STF-HD molecules also make contacts with symmetry-related DNA molecules in the crystal. Aiming to obtain further mechanistic insights, we carried out structure-based mutagenesis to identify key residues in STF-HD that are essential for DNA binding and STF function. We chose the *MtAS2* promoter region, which is an important binding target of STF for leaf-blade development *in vivo* (Zhang *et al.*, 2014 ▸). We also took advantage of the *N. sylvestris* bladeless mutant *lam1* to perform complementation assays, in which STF or STF mutants were all driven by the same STF promoter. When STF was driven by the STF promoter, the *lam1* mutant was completely complemented (Zhang *et al.*, 2014 ▸).

**Figure 8:**
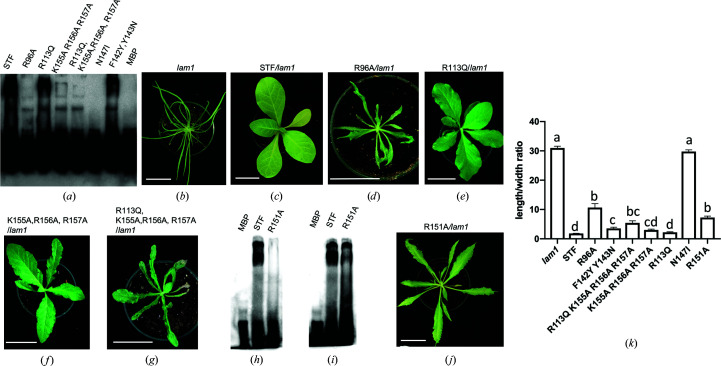
Key residues of STF-HD for DNA binding and *in vivo* function. (*a*) EMSA showing that mutations in STF-HD affect its ability to bind to the *MtAS2* promoter sequence *in vitro*. (*b*)–(*g*) Phenotypes of *N. sylvestris* plants: *lam1* mutant (*b*), plants complemented with wild-type STF:STF corresponding to the WT phenotype (*c*), mutants STF:STF-R96A (*d*), STF:STF-R113Q (*e*), STF:STF-K155A/R156A/R157A (*f*), STF:STF-R113Q/K155A/R156A/R157A (*g*). (*h*) EMSA showing that the R151A mutation nearly abolished the binding of STF to the TGA sequence (GCAAATCTATGATCTATTCAAG). (*i*) EMSA showing that the R151A mutation still retained its binding to the TAAT sequence (GCAAATTAATTATTTATTAAAG). (*j*) Phenotype of a *lam1* mutant *N. sylvestris* plant complemented with STF:STF-R151A. (*k*) Leaf length/width ratios of the largest leaves of six-week-old plants. At least ten independent lines were analyzed for each construct. Statistical analyses were performed using one-way ANOVA followed by a Tukey’s test (*p* < 0.05).

Firstly, we set out to probe the residues on STF-HD that are involved in TGA recognition. When we replaced Arg96 with an alanine, we found that the R96A mutation abolished the binding of STF-HD to *MtAS2* promoter DNA in the EMSA study (Fig. 8 ▸ *a*). We also found that the R96A mutation greatly reduced *lam1* mutant complementation in plant growth (Figs. 8 ▸ *d*, 8 ▸ *k* and 9 ▸ *g*). These observations support Arg96 playing an essential role in STF in binding and repressing its promoter DNA *in vivo*. We previously showed that the N147I single mutation abolished the DNA binding of STF-HD containing both TGA and TAAT sequences in the EMSA assays, and also abolished the *lam1* complementation *in planta* (Zhang *et al.*, 2014 ▸; Figs. 8 ▸ *a* and 8 ▸ *k*). When testing the impact of the R151A mutation on the DNA binding of STF-HD using EMSA, we found that the mutation abolished its binding to a sequence containing only TGA motifs (GCAAATCTATGATCTATTCAAG), while it retained its binding to a sequence containing TAAT motifs (GCAAATTAATTATTTATTAAAG) (Figs. 8 ▸ *h* and 8 ▸ *i*). However, the R151A mutant displayed a significant loss of STF function *in planta*, leading to a severe defect in leaf-blade growth (Figs. 8 ▸ *j*, 8 ▸ *k* and 9 ▸ *h*). These data suggest that both the affinity and precise structural conformation of DNA binding are essential for STF function *in vivo*.

**Figure 10:**
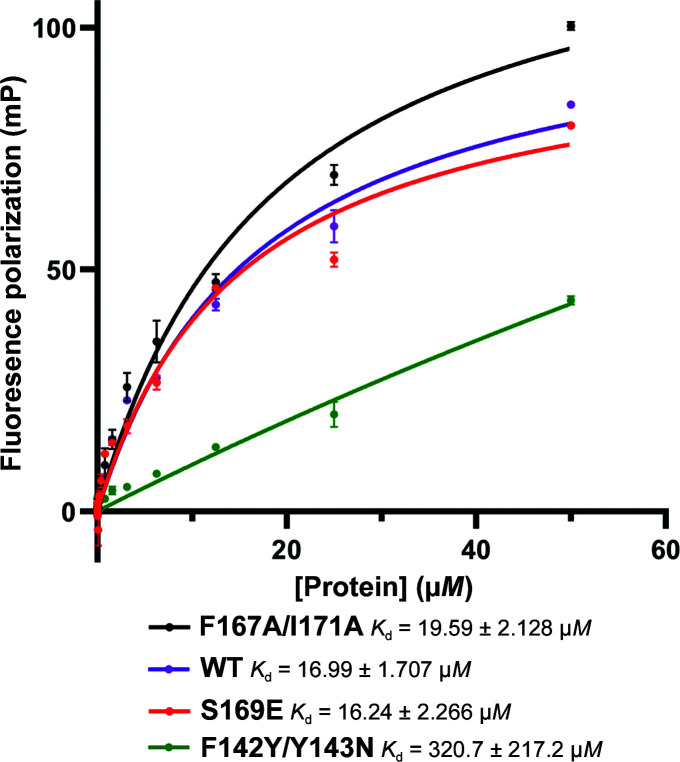
Fluorescence polarization assay of DNA binding by STF-HD proteins. Fluorescent polarization is represented by millipolarization units (mP). The *K*_d_ values for each of the STF-HD proteins were calculated by nonlinear regression and standard deviations from triplicate values. The F142Y/Y143N mutant had a higher *K*_d_ value, indicating weaker binding to the DNA probe.

**Figure 11:**
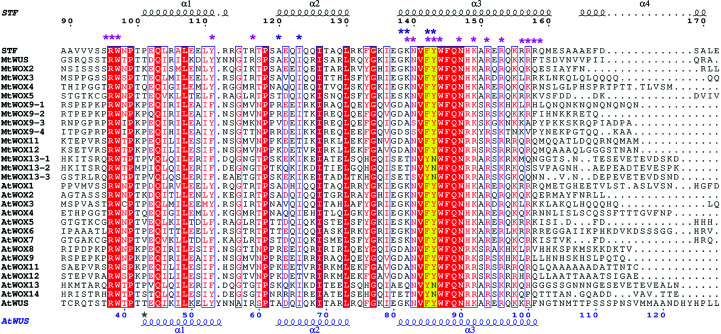
Structure-based sequence alignment of selected WOX HD domains. A structure-based sequence alignment of various STF-HD orthologs was created using the crystal structure of STF-HD as the template. Secondary structures and residue numbering above the alignment correspond to STF^85–190^ and those for WUS-HD are shown in blue at the bottom. Sequence alignment was performed with the *SSM* server (Krissinel & Henrick, 2004 ▸) and the figure was created with *ESPript* (Gouet *et al.*, 2003 ▸). Residues involved in DNA binding are indicated with purple asterisks and residues that constitute the docking platform at the dimer interface are indicated with blue asterisks.

Next, we carried out site-directed mutagenesis on STF-HD, focusing on both the observed protein–protein interactions and DNA binding that are associated with HDA′ and HDB′. We first set out to evaluate the significance of the C-terminal helix α4 of STF-HD for DNA binding and STF function. We generated two mutants (S169E and F167A/I171A) of STF-HD, aiming to perturb the protein–protein interactions since these residues are observed to be involved in bridging the four STF-HD protomers in the structure (Fig. 3 ▸). We found that neither mutant showed a significant decrease in the DNA-binding affinity in our FP assays (Fig. 10 ▸) or caused a significant defect in the leaf growth of plants (not shown). We subsequently carried out mutations of the two aromatic residues Phe142 and Tyr143 that are located at the N-terminus of helix α3. Both residues in HDA and HDB are involved in interactions with helix α4 of HDA′ (Fig. 3 ▸). Phe142 and Tyr143 are highly conserved among many WOX HD family members; however, certain species contain YN at the equivalent positions (Fig. 11 ▸). We found that although the F142Y/Y143N double mutant showed slightly reduced DNA binding in the EMSA assay (Fig. 8 ▸ *a*), it displayed a significant decrease in DNA-binding affinity in our FP studies (Fig. 10 ▸). Accordingly, reduced *lam1* complementation and defects in leaf-blade growth were observed (Figs. 8 ▸ *k* and 9 ▸ *f*).

In addition, mutations were generated to probe the observed minor-groove DNA binding of helices α3 of HDA′ and HDB′. Triple alanine substitutions of the positive charge cluster on the C-terminus of helix α3, KRR/AAA (155–157), reduced the DNA binding of STF-HD in EMSA, while a combination of KRR/AAA and R113Q mutations greatly decreased the DNA binding (Fig. 8 ▸ *a*). Both mutants displayed defects in plant leaf-blade growth (Figs. 8 ▸ *f*, 8 ▸ *g*, 9 ▸ *c* and 9 ▸ *d*). However, the single mutation R113Q affected neither the DNA binding nor STF function *in vivo* (Figs. 8 ▸ *a*, 8 ▸ *e* and 9 ▸ *b*), which is consistent with the observation that Arg113 does not directly interact with the DNA in the structure.

### 3.4. Comparison with WUS-HD   

When we compared the structures of STF-HD with those recently reported for WUS-HD (Sloan *et al.*, 2020 ▸), we found that the cores of both proteins adopt nearly identical conformations, with an r.m.s.d. of 0.77 Å over 61 equivalent C^α^ atoms (Fig. 2 ▸ *c*). Since apo WUS-HD displays an identical core structure to that observed in its DNA complexes (Sloan *et al.*, 2020 ▸), it is also likely that STF-HD may not undergo significant conformational changes upon binding DNA. The three helices and the two connecting loops in both STF-HD and WUS-HD superimpose well. In the WUS-HD structure, the unique Tyr54 located in helix α1 leads to a slight distortion of its end and a slightly longer connecting loop I between helices α1 and α2 when compared with the structure of a canonical HD protein, Engrailed (PDB entry 3hdd; Sloan *et al.*, 2020 ▸). However, STF-HD does not contain a tyrosine at the equivalent position; instead, it contains Arg112, albeit with a disordered side chain. The major differences between the structures are observed in the N-terminal arms and the C-termini. The N-terminal arm in STF-HD is slightly longer, while the length of helix α3 varies in a context-dependent manner in both the STF-HD and WUS-HD structures. The longest α3 helices are observed in STF HDA and the WUS-HD–TGAA complex (PDB entry 6ryd); however, helix α3 in STF HDA is one helical turn longer (Fig. 2 ▸ *c*). The structure of STF HDA contains an additional short helix α4 at its C-terminus, which is absent from all the WUS-HD structures.

**Figure 12:**
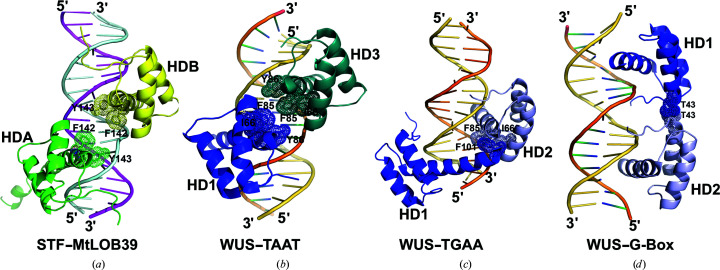
Comparison of DNA binding by STF HDA and HDB with WUS-HD dimers. (*a*) STF HDA (green) and HDB (yellow). The nonpolar residues buried at the interfaces are shown as sticks, with dotted envelopes indicating the van der Waals radius. STF HDA and HDB are more than 8 Å apart and do not contact each other directly. (*b*) WUS-HD dimer (blue and teal) bound to the TAAT motif (PDB entry 6ryl). WUS HD1 is bound at the TAAT core, while WUS HD3 is bound at a neighboring location without base-specific interactions. (*c*) WUS-HD dimer (blue and light blue) bound to the TGAA repeat (PDB entry 6ryd). (*d*) WUS-HD dimer (blue and light blue) bound to the G-box motif (PDB entry 6ryi).

Homodimers of WUS-HD were observed in all three DNA complex structures (TGAA repeat, G-box and TAAT motif) with various dimerization interfaces involving protein–protein interactions in a context-dependent manner (Fig. 12 ▸; Sloan *et al.*, 2020 ▸). In the current structure of the complex of STF-HD and DNA, four molecules of STF-HD are bound on the same DNA, clustering on the two TGA motifs that are arranged in an inverted repeat orientation and separated by five base pairs (Figs. 2 ▸ *b* and 4 ▸). In the asymmetric unit of the crystal, HDA and HDB each specifically recognize a TGA motif. In each protomer, helix α3 is inserted in the major groove, while the N-terminal arm embraces the DNA in the minor groove (Fig. 2 ▸ *a*). This overall DNA-binding pattern of HDA and HDB is similar in part to that observed in the structure of WUS-HD in complex with a TAAT motif (PDB entry 6ryl). In this structure, three WUS-HD molecules were bound on the DNA, with two recognizing the TAAT sequence from the opposite sides of the DNA, while the third binds the DNA in a promiscuous manner without clearly defined protein–base interactions and with higher flexibility, as indicated by high *B* factors. This third WUS HD3 forms a homodimer with WUS HD1, with tight protein–protein associations through hydrophobic interactions involving Ile66, Phe85 and Tyr86 from both WUS HDs, in a head-to-head abutting manner (Sloan *et al.*, 2020 ▸). The overall arrangement of the DNA binding of STF HDA and STF HDB is similar to that observed in the WUS HD1 and WUS HD3 dimer (Figs. 12 ▸ *a* and 12 ▸ *b*). However, there are no direct protein–protein interactions between the two STF HDs. Instead, the equivalent hydrophobic residues Ile123, PheF142 and Tyr143 in STF HDA and STF HDB sandwich the C-terminal helix α4 of STF HDA′ at the tetramer interface (Figs. 2 ▸ *b* and 3 ▸). These residues in STF HDB′ are associated with helix α4 of STF HDA.

**Figure 13:**
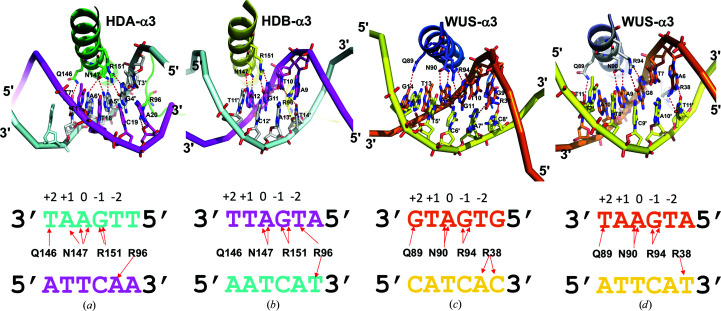
Comparison of major-groove base recognition between STF-HD and WUS-HD. (*a*) TGA recognition by STF HDA helix α3 (green). (*b*) TGA recognition by STF HDB helix α3 (yellow). (*c*) G-box binding by WUS-HD helix α3 (blue). (*d*) TGAA recognition by WUS-HD helix α3 (light blue). The diagrams below each cartoon illustrate the DNA base contacts made by each HD, with red arrows indicating hydrogen bonds.

The overall mechanism of TGA readout by STF HDA and STF HDB is similar to that found in the structures of WUS-HD bound to TGAA and G-box repeats (Fig. 13 ▸; Sloan *et al.*, 2020 ▸). Located on helix α3, Asn147, which is equivalent to the canonical Asn51 in standard HD numbering (Bürglin & Affolter, 2016 ▸), specifies an adenine (position 0), while Arg151 specifies a guanine at position −1 from the adenine via hydrogen bonding in the major groove. Both Asn147 and Arg151 are strictly conserved among the WOX family members (Fig. 11 ▸), with the corresponding residues in WUS-HD being Asn90 and Arg94, respectively (Fig. 13 ▸). Unique to STF-HD, Asn147 in HDA contacts both the adenine at position 0 and another adenine at position +1, while Asn90 in WUS-HD only contacts the adenine at position 0. In all three structures of WUS-HD, the +1 position of the DNA motif made no hydrogen bonds to the protein (Sloan *et al.*, 2020 ▸). In addition, while Arg96 of STF HDA contacts A20′ at position −2 in the reverse DNA strand, Arg96 of STF-HDB contacts T10 at position −2 as well as T14′ at position −3 in the reverse strand (Figs. 13 ▸ *a* and 13 ▸ *b*). In comparison, the corresponding Arg38 in WUS-HD only contacts the bases at the −2 and/or −3 positions in the reverse strand (Figs. 13 ▸ *c* and 13 ▸ *d*).

In all three DNA complex structures of WUS-HD, the base-recognition helices α3 are exclusively found to interact with the DNA in the major grooves. Unique to STF-HD, the helices α3 in both HDA′ and HDB′ make contacts with the backbone and bases of the DNA in the minor grooves, which are also important for DNA binding and STF function.

## 4. Discussion   

The crystal structure of STF-HD in complex with its target promoter DNA displayed a unique STF-HD tetramer clustered on the two closely neighboring TGA motifs, although the sequence also contains TAAT motifs. Analyzing the known available genomic promoter regions that WUS and STF bind indicates that this sequence feature is quite common (Supplementary Table S3), suggesting that the observed tetrameric binding of HD could possibly be a shared mechanism. In the current structure, two STF-HDs specifically interact with the TGA motifs via the recognition helices α3 inserted in the major grooves, while two additional STF-HDs interact with the DNA by inserting their α3 helices into the minor grooves.

The binding specificity of each TGA motif by STF-HD is mainly provided by two conserved residues Asn147 and Arg151 located on helix α3 that recognize the G/A step in the major groove. The conserved Arg96 in the N-terminal arm embraces the DNA in the minor groove, providing binding affinity. This DNA readout mode closely mimics those observed in other HD structures, including WUS-HD (Sloan *et al.*, 2020 ▸; Lee *et al.*, 2018 ▸; Li *et al.*, 1995 ▸; Passner *et al.*, 1999 ▸; LaRonde-LeBlanc & Wolberger, 2003 ▸). Our mutagenesis studies showed that mutations of either Arg96 or Asn147 abolished the DNA binding of STF-HD *in vitro* and its function *in vivo*, leading to leaf-growth defects (Figs. 8 ▸ and 9 ▸; Zhang *et al.*, 2014 ▸). Interestingly, although the R151A mutant abolished STF-HD binding to a DNA sequence containing only TGA motifs, it retained DNA binding to the promoter sequence containing TAAT motifs (Figs. 8 ▸ *h* and 8 ▸ *i*). Nevertheless, R151A mutant STF is functionally defective *in vivo*, with a loss of promoter repression, and caused a defect in leaf growth. This suggests that the precise structural conformation of the complex of STF-HD and the bound DNA determines its promoter repressive activity *in vivo*, rather than the DNA-binding affinity alone.

STF-HD clamps onto the DNA as a tetramer over nearly the entire bound surface (Fig. 2 ▸ *d*), in contrast to just a portion of the bound DNA surface as observed in other structures. Besides DNA binding, the STF-HD tetramer is bridged through the C-terminal α4 helices from STF HDA and its crystallographically related HDA′. Although we could not exclude the possibility of the observed tetrameric association of STF-HD being a crystallization artifact caused by crystal packing, our mutagenesis and functional studies suggest that the observed DNA interactions of HDA′ and HDB′ are functionally significant. The α3 helices in both HDA′ and HDB′ interact with the DNA in the minor grooves via the C-termini, which has not previously been observed. Mutations of charged residues at this location reduced the DNA binding and the ability of STF to rescue the *lam1* mutant (Figs. 8 ▸ *a*, 8 ▸ *f*, 8 ▸ *g*, 9 ▸ *c* and 9 ▸ *d*) *in planta*, displaying defects in leaf-blade growth.

Protein–protein interactions could play an important role in determining how HDs bind DNA. It has been shown that DNA binding by WUS-HD is largely dependent on the homodimerization of WUS-HDs, although apo WUS-HD appeared to be a monomer in solution (Sloan *et al.*, 2020 ▸). STF-HD also appears to be monomeric in the absence of DNA, and the tetrameric association of STF-HD is driven by DNA binding (Fig. 1 ▸). The protein–protein interactions in the current structure involve a hydrophobic surface patch that is located on helices α2 and α3, which serves as a common docking site for the α4 helices in HDA and HDA′ at the tetramer interface (Fig. 3 ▸). This hydrophobic patch is also found in the WUS-HD structures: it is comprised of Ile66, Phe85 and Tyr86 and is involved in two different modes of homodimerization (Sloan *et al.*, 2020 ▸). In the structure of the WUS-HD–TAAT complex this hydrophobic surface is involved in extensive interactions between two WUS-HDs, one bound at the TAAT core and the other bound on a less defined juxtaposition. Although the overall orientation and arrangement of this DNA-bound WUS-HD dimer is in part similar to that found in STF HDA and STF HDB bound with DNA, there are no direct protein–protein interactions between the two STF promoters (Figs. 2 ▸ *a* and 12 ▸ *a*). The other mode of homodimerization of WUS-HD involving this hydrophobic patch is found in the structure of the WUS-HD–TGAA complex. The hydrophobic patch from one protomer serves as a docking site for the C-terminus of the recognition helix α3 in the other, involving the aromatic residue Phe101 (Fig. 12 ▸ *c*). This dimerization mode of WUS-HD is similar to those observed in STF HDA–HDA′, HDB–HDA′ and HDA–HDB′ protein–protein associations, which however instead involve helices α4 (Fig. 2 ▸ *b*). The F142Y/Y143N double mutant at the STF-HD tetramer interface displayed a significant reduction in DNA-binding affinity (Fig. 10 ▸). These observations are similar to those found in DNA-binding studies of WUS-HD. Specifically, an F101A mutation at the C-terminus of helix α3 in WUS-HD only led to a small reduction in binding affinity, compared with a >20-fold reduction for the I66A and F85A mutants that are located on the docking patch at the dimer interface (Sloan *et al.*, 2020 ▸). It was shown that although the F101A mutant still bound DNA with reasonable affinity, it disrupted the homodimerization of the WUS-HD in the DNA complex, suggesting that this weak protein–protein interaction is nevertheless functionally important. Interestingly, we did not observe significant defects of the S169E and F167A/I171A mutants in *in vitro* DNA binding and leaf development *in planta*. This might be due to the very small protein–protein interface involved, the perturbation of which may not be sufficient to disrupt the tetrameric association of STF-HD that seems to be largely scaffolded by DNA binding. Despite the fact that helix α4 is not conserved at the sequence level and is absent from WUS-HD, a similar structure could exist among WOX family members (Fig. 11 ▸). In fact, the protein construct of WUS-HD used in the structural analyses contained only residues up to the end of helix α3 (residues 34–103; Fig. 11 ▸), lacking helix α4. It has yet to be shown whether the tetrameric DNA-binding mode mediated by helix α4 is a shared common feature of other WOX family members with specific promoter sequences.

The WOX family has been phylogenetically divided into the WUS/modern clade, the WOX9/intermediate clade and the WOX13/ancient clade, with transcriptional repression activity in the WUS clade and activation activity in the WOX9 and WOX13 clades (Lin, Niu, McHale *et al.*, 2013 ▸; Dolzblasz *et al.*, 2016 ▸; Wu *et al.*, 2019 ▸; van der Graaff *et al.*, 2009 ▸). WUS clade WOX family proteins also require TPL corepressors for function (Causier *et al.*, 2012 ▸). The C-terminal domains of WUS and STF contain a WUS box and an ethylene response factor-associated amphiphilic repression (EAR)-like motif, which have been shown to be crucial for the recruitment of TPL corepressors (Causier *et al.*, 2012 ▸; Zhang *et al.*, 2014 ▸). It has previously been shown that the oligomerization of EAR motifs in certain repressors could enhance the binding affinities/avidities of TPL proteins through multivalent inter­actions (Martin-Arevalillo *et al.*, 2017 ▸). The tetramerization of STF-HD could therefore potentially enhance its association with TPL, and the resulting more stable repressor complex could be important for regulating key plant developmental programs.

## Supplementary Material

PDB reference: STENOFOLIA homeodomain, 6wig

Supplementary Tables. DOI: 10.1107/S205979832100632X/qh5070sup1.pdf

## Figures and Tables

**Figure 7:**
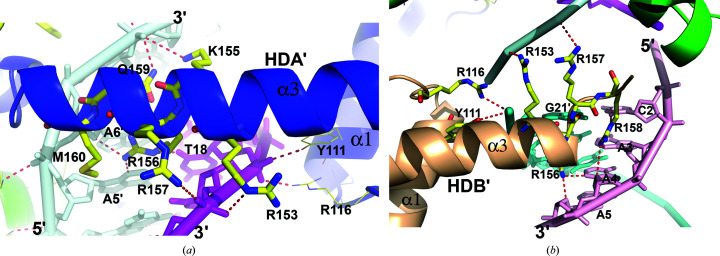
DNA minor-groove interactions of the α3 helices. (*a*) HDA′. (*b*) HDB′. The color scheme is the same as in Fig. 2 ▸(*b*). Contacting residues on the α3 helices are shown as sticks. Hydrogen bonds are shown as red dashed lines.

**Figure 9:**
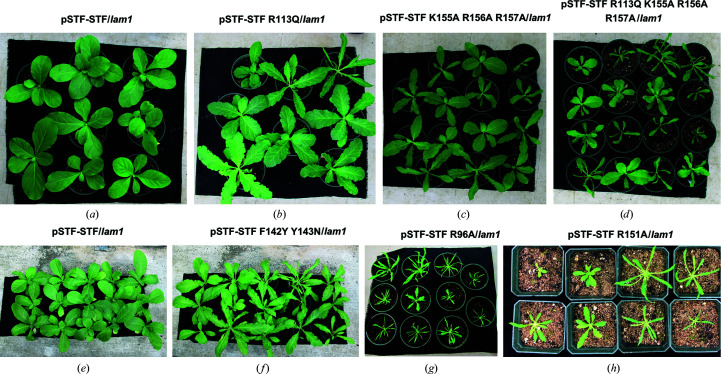
Phenotypes of *lam1* transformed with STF or STF mutants driven by the STF promoter. (*a*–*d*) Transgenic *lam1* plants complemented with STF:STF (*a*), STF:STF R113Q (*b*), STF:STF K155A/R156A/R157A (*c*) and STF:STF R113Q/K155A/R156A/R157A (*d*) constructs at four weeks old. (*e*, *f*) Transgenic *lam1* plants complemented with STF:STF (*e*) and STF:STF F142Y/Y143N (*f*) constructs at five weeks old. (*g*) Transgenic *lam1* plants complemented with the STF:STF R96A construct at four weeks old. (*h*) Transgenic *lam1* plants complemented with the STF:STF R151A construct at four weeks old.

**Table 1:** Crystallographic data and statistics Values in parentheses are for the highest resolution shell.

	SeMet STF_3M-HD†, peak	Native STF-HD
Data collection		
Beamline	19-ID, APS	19-ID, APS
Wavelength (Å)	0.97918	0.97935
Space group	*P*2_1_	*P*2_1_
*a*, *b*, *c* (Å)	46.3, 49.0, 70.0	48.1, 49.5, 69.8
α, β, γ (°)	90, 105.9, 90	90, 106.5, 90
Resolution (Å)	50.00–2.50 (2.59–2.50)	50.00–2.10 (2.18–2.10)
Total reflections	55241	142294
Unique reflections	10086 (794)	17554 (1431)
Multiplicity	5.5 (3.6)	8.1 (5.8)
Completeness (%)	94.1 (74.0)	95.2 (77.7)
〈*I*/σ(*I*)〉	14.2 (2.5)	23.5 (1.9)
*R*_merge_ ‡ (%)	13.0 (48.6)	8.0 (66.2)
CC_1/2_ (%)	95.4 (81.6)	(81.4)
Refinement statistics		
Resolution range used (Å)		46.1–2.1
No. of reflections used		17488
*R*_work_/*R*_free_ § (%)		19.4/25.0
R.m.s.d., bond lengths (Å)		0.010
R.m.s.d., bond angles (°)		1.214
No. of atoms		
Protein		1285
Ligand		902
Water		44
Average *B* (Å^2^)		
Protein		65.0
Ligand		55.0
Water		46.5
Ramachandran values		
Preferred regions (%)		97.7
Allowed regions (%)		2.3

† STF_3M-HD is triple-mutant (L107M/L110M/L130M) STF^85–190^.

‡ *R*_merge_ = \textstyle \sum_{hkl}\sum_{i}|I_{i}(hkl)- \langle I(hkl)\rangle|/\textstyle \sum_{hkl}\sum_{i}I_{i}(hkl).

§ *R*_free_ was calculated using 5% of the data.
