# Supplementary material for: Structure of the unique tetrameric STENOFOLIA homeodomain bound with target promoter DNA
Source: Acta Crystallogr D Struct Biol. 2021 Jul 29;77(Pt 8):1050–63. doi: 10.1107/S205979832100632X (PMC8329861; doi:10.1107/S205979832100632X)
Supplement: Supplementary file 1 [file d-77-01050-sup1.pdf]

Acta Cryst. (2021). D**77**, https://doi.org/10.1107/S205979832100632X Supporting information

STRUCTURAL BIOLOGY

Volume 77 (2021)

Supporting information for article:

Structure of the unique tetrameric STENOFOLIA homeodomain bound with target promoter DNA

Prabhat Kumar Pathak, Fei Zhang, Shuxia Peng, Lifang Niu, Juhi Chaturvedi, Justin Elliott, Yan Xiang, Million Tadege and Junpeng Deng

Table S1 Primers used in this study.

Probes for EMSA

MtAS2-F TGGATTTAATAATGAAGTGACA

MtAS2-R TGTCACTTCATTATTAAATCCA

MtLOB39-F GCAAATTAATGATTTATTCAAG

MtLOB39-R CTTGAATAAATCATTAATTTGC

TGA-F GCAAATCTATGATCTATTCAAG

TGA-R CTTGAATAGATCATAGATTTGC

TAAT-F GCAAATTAATTATTTATTAAAG

TAAT-R CTTTAATAAATAATTAATTTGC

Mutations

L107MF AACAGTTAAGAGCAaTGGAAGAATTG

L107MR CAATTCTTCCATTGCTCTTAACTGTT

L110MF GCACTGGAAGAAaTGTATAGAAGAGG

L110MR CCTCTTCTATACATTTCTTCCAGTGC

L130MF CAAATAACTGCCCAGaTgAGAAAATTTGG

L130MR CCAAATTTTCTCATCTGGGCAGTTATTTG

L107110MF AACAGTTAAGAGCAaTGGAAGAAaTGTATAGAAGAGG

L107110MR CCTCTTCTATACATTTCTTCCATTGCTCTTAACTGTT

N147IF TCTATTGGTTTCAGATTCACAAAGC

N147IR GCTTTGTGAATCTGAAACCAATAGA

FY-F AGGCAAAAATGTTTACTATTGGTTTCAGAA

FY-R TTCTGAAACCAATAGTAAACATTTTTGCCT

R/Q-F GAAGAATTGTATAGACAAGGAACAAGAACAC

R/Q-R GTGTTCTTGTTCCTTGTCTATACAATTCTTC

KRR-F CAGAGAAAGGCAAGCAGCAGCGCGTCAAATGGAATC

KRR-R CAGAGAAAGGCAAGCAGCAGCGCGTCAAATGGAATC

R151A-F CAGAATCACAAAGCCAGGCAAAGGCAAAAACGACGGC

R151A-R GCCGTCGTTTTTGCCTTTGCCTGGCTTTGTGATTCTG

Protein expression

STF-N85-NcoI-F aaccatgggaAATAATCCATCTGCAGCAGTTGTG

STF-N190-HindIII-R aaaagcttTCAGTTTTTAGTGTGTTCAACTTCAAACAC

Complementation

STF-F-GW GGGGACAAGTTTGTACAAAAAAGCAGGCTTCATGTGGATGGTGGGTTACAAT

STF-R-GW

GGGGACCACTTTGTACAAGAAAGCTGGGTCTCAGTTTTTCAAGGGAAGAAACT

Table S2 Phenotypes of complementation assays in *Nicotiana sylvestris* using *lam1* bladeless mutant

STF proteins	Length/width ratio of the biggest leaf					plants examined
	>25	10-25	5-10	2.5-5	<2.5	
STF	0	0	0	0	12	12
lam1	10	0	0	0	0	10
STF R96A	0	7	2	2	0	11
STF R113Q	0	0	0	3	8	11
STF K155A R156A R157A	0	0	0	8	3	11
STF R113Q K155A R156A R157A	0	0	7	4	1	12
STF R151A	0	0	10	3	0	13
STF N147I	10	0	0	0	0	10
STF F142Y Y143N	0	0	2	10	2	14

Plants were measured after 6 weeks of growth in the greenhouse. Leaves with length/width ratio over 25 were without any complementation, while leaves with length/width less than 2.5 were complemented to that level of wild type plants.

Table S3 Known target promoters for AtWUS and STF

Known AtWUS targets:

AtCLV3 promoter (https://doi.org/10.1073/pnas.1607669113)

CCGTTGGGAAAATTTATTAGTACGTTTTCAATTGTCAATGCAAATAATTAATGGATGTGATAGTCACAATTAAACATACAATAATAAAAATGATGATGATGATTCGATGATGTGGTGGGAAGGATAAATTAAACCGACTTTGGGGCA

AtAG promoter (https://doi.org/10.1016/S0092-8674(01)00384-1)

AGAGACAAAAATTATAAGGATACTAAATTGTTCATATTTCTTGGAAGTAAAAAGATTAATGATCACTGAATAAATAGATTTGGCATAGAAGTATAGC

ARR7 promoter (https://doi.org/10.1038/nature04270)

TTTCATGTTTGACACTTATTTAGGGATCGTATAATATACGTTTTTTAATTGATTTGAAACCAAATAACATGTACTATTGCCTTAGTCCTCTTGCAATTCGTTTAGAAGATGTTATGATTGAGACAAAA

AtHEC1 promoter (https://doi.org/10.1016/j.devcel.2014.01.013)

TTGATAATCTTTTACTCCAGCTATTATATGAAACCTAATTGACCTTTTTCACATACAAAAGTGTTGATTATGAAATGAATACAATAATAATGGTG

STF targets:

MtAS2 promoter (https://doi.org/10.1105/tpc.113.121947)

CGCATTAATGGATCAGTTCTTATTTCCTTTTATAATTTTTTATTACCATTATTTTATTATTTTACAACATAGTAATAACAAACAAAAACATGGTGAATGCATCACAGAAATTAATGGTAGTGTGAAAGAGAGAGAGACAGA

MtWOX9 promoter (https://doi.org/10.1111/nph.16934)

ATATATTCCATATTTATTATGATATATAAACTTTTCATCTTACATTTTAATATGTTAAATAAATTCTTTATACTACTAATAATGAATTATTGTTATTATTGCAATTATTA

MtLOB39 promoter (this study)

TGTAGAATATTTTTTTTATCTTTATGAATGAAATTGTATTCAAATTAATGATTTATTCAATTTTTTAATACAGTATTAATTATCACTTTCAATTATACATTTTAATTAATATTAATTT
